# Maternal isolated hypothyroxinemia in the first trimester is not associated with adverse pregnancy outcomes, except for macrosomia: a prospective cohort study in China

**DOI:** 10.3389/fendo.2023.1309787

**Published:** 2023-12-14

**Authors:** Jing Du, Linong Ji, Xiaomei Zhang, Ning Yuan, Jianbin Sun, Dan Zhao

**Affiliations:** ^1^ Department of Endocrinology, Peking University People’s Hospital, Beijing, China; ^2^ Department of Endocrinology, Peking University International Hospital, Beijing, China

**Keywords:** isolated hypothyroxinemia, pregnancy outcome, macrosomia, first trimester, prospective cohort study

## Abstract

**Objective:**

Insufficient thyroid hormone levels during pregnancy, especially in the first trimester, adversely affect maternal and fetal health. However, the impact of isolated hypothyroxinemia (IH) on adverse pregnancy outcomes remains controversial. Therefore, this study aimed to investigate the association between IH during the first trimester and adverse pregnancy outcomes.

**Methods:**

This prospective cohort study included 1236 pregnant women. Thyroid-stimulating hormone and free thyroxine levels were measured before 13 weeks of gestation. Logistic regression analysis and the Cochran-Armitage trend test were used to assess the association between IH in the first trimester and adverse pregnancy outcomes.

**Results:**

IH during the first trimester was associated with an increased risk of macrosomia. After adjusting for confounding factors, including age, body mass index, parity, abnormal pregnancy history, fasting blood glucose, and total cholesterol, multivariate logistic regression analysis showed that IH in the first trimester remained an independent risk factor for macrosomia. In addition, the risk of macrosomia increased with IH severity. However, no significant relationship was found between IH during the first trimester and gestational diabetes mellitus, hypertensive disorders of pregnancy, spontaneous abortion, premature rupture of membranes, placental abruption, oligohydramnios, premature delivery, fetal distress, or low birth weight.

**Conclusion:**

IH during the first trimester did not increase the risk of adverse pregnancy outcomes, except for macrosomia.

## Introduction

Thyroid hormones are essential for maintaining normal pregnancy and fetal development (1). Insufficient thyroid hormone levels during pregnancy, especially in the first trimester, adversely affect maternal and fetal health and the neurodevelopment of offspring (2–4). Thyroid hormone deficiency during pregnancy includes overt hypothyroidism, subclinical hypothyroidism, and isolated hypothyroxinemia (IH). According to the 2017 Guidelines of the American Thyroid Association (5), IH during pregnancy is defined as a free thyroxine (FT4) concentration below the 2.5th-5th percentile of the pregnancy-specific reference range, in conjunction with a normal thyroid-stimulating hormone (TSH) concentration.

Numerous studies have demonstrated that overt and subclinical hypothyroidism are associated with an increased risk of adverse pregnancy outcomes, including pregnancy loss, premature delivery, gestational hypertension, and gestational diabetes mellitus (6–8). The incidence of IH during pregnancy has been gradually increasing (9). However, the impact of IH on adverse pregnancy outcomes has not been extensively studied, and the results remain controversial (10). Furthermore, there is currently no consensus regarding the use of levothyroxine (LT4) therapy for IH during pregnancy (5, 11). Therefore, this prospective cohort study aimed to investigate the influence of IH during early pregnancy on adverse pregnancy outcomes to provide a reference for clinical treatment.

## Materials and methods

### Study population

**Figure 1:**
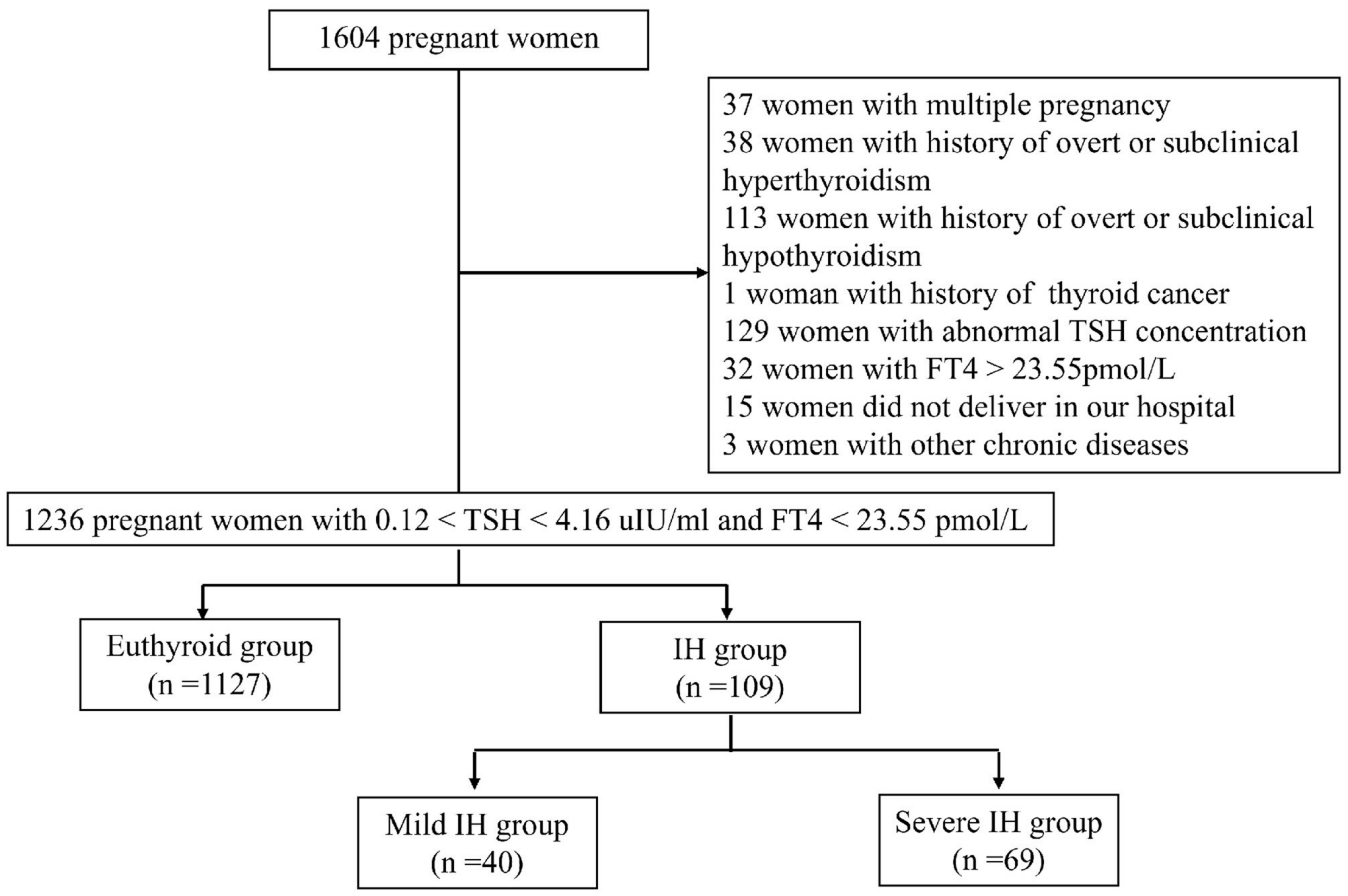
Flow chart depicting participants’ selection process.

This was a single-centre, prospective cohort study conducted at the Department of Obstetrics and Gynaecology of Peking University International Hospital. Between October 2016 and April 2018, 1604 pregnant women voluntarily participated in this study. The inclusion criteria were as follows: (1) age ≥18 years; (2) residency in Beijing for more than 5 years; (3) singleton pregnancy; (4) gestational age < 13 weeks determined by last menstrual period and human chorionic gonadotropin; (5) euthyroidism or IH in the first trimester; and (6) planning to undergo examination and give birth at our hospital. The exclusion criteria were as follows: (1) multiple pregnancy; (2) history of thyroid diseases, including overt or subclinical hypothyroidism, hyperthyroidism, and thyroid cancer; (3) treatment with any drug affecting thyroid function, such as LT4, methimazole, propylthiouracil, and amiodarone; (4) presence of liver and kidney, respiratory, cardio-cerebrovascular, haematological, or autoimmune diseases (e.g., antiphospholipid syndrome, systemic lupus erythematosus), and other tumours; (5) thyroid dysfunction (abnormal TSH concentration and/or elevated FT4 concentration beyond the 97.5th percentile of the pregnancy-specific reference range) in the first trimester; and (6) did not deliver in our hospital. Finally, a total of 1236 pregnant women were included in this study (**Figure 1**). This study was approved by the Ethics Committee of Peking University International Hospital. All participants signed the informed consent.

### Data collection

Demographic and medical data, including age, parity, gestational age, medical history, abnormal pregnancy history (miscarriage, premature birth, stillbirth, birth defects, etc.), and medications, were collected from the participants via a medical questionnaire. Trained nurses measured the participants’ height and weight. Body mass index (BMI) was calculated as weight (kg) divided by height squared (m^2^).

Fasting venous blood samples were collected from women during early pregnancy to examine fasting blood glucose (FPG), total cholesterol (TC), triglyceride (TG), high-density lipoprotein cholesterol (HDL-C), low-density lipoprotein cholesterol (LDL-C), serum iron (Fe), and ferritin levels. Levels of TSH and FT4 were measured using a Roche Cobas Elecsys 601 analyzer (Roche, Basel, Switzerland).

All women received regular follow-ups throughout pregnancy. Pregnancy and delivery information, including pregnancy loss, premature delivery, and other pregnancy complications, as well as neonatal data (sex, weight, height, and health status), were recorded.

### Definitions

In our previous study (12), we established reference ranges for thyroid function during pregnancy at Peking University International Hospital, according to the standards of the American Institute of Clinical Biochemistry. In the first trimester, the reference intervals for gestational TSH and FT4 were 0.12-4.16 uIU/mL and 13.36-23.55 pmol/L (both within the 2.5th and 97.5th percentiles), respectively. In the current study, IH was defined as an FT4 level below the 5th percentile and a TSH level within the trimester-specific reference range. The 5th percentile of FT4 in the first trimester was 13.80 pmol/L. Euthyroidism was defined as an FT4 level between the 5th and 97.5th percentiles with a normal TSH level. Furthermore, mild IH was defined as FT4 levels between the 2.5th and 5th percentiles, whereas severe IH was defined as FT4 levels below the 2.5th percentile.

Adverse pregnancy outcomes in this study included gestational diabetes mellitus (GDM), hypertensive disorders of pregnancy (HDP), spontaneous abortion, premature rupture of membranes (PROM), placental abruption, oligohydramnios, premature delivery, fetal distress, macrosomia, and low birth weight. The definitions for these outcomes were based on previous studies (13, 14). Placental abruption was defined as the premature separation of a normally implanted placenta from the uterine wall (15). The definition of oligohydramnios was a maximum vertical pocket measuring less than 2 cm (15).

### Statistical analysis

Statistical analyses were performed using SPSS software (version 20.0; IBM, Armonk, NY, USA). The normality of continuous variables was assessed using the Shapiro-Wilk test. Continuous variables are presented as either mean ± standard deviation or median (interquartile range), depending on their distribution. Categorical variables are presented as absolute numbers and percentages. Differences between the two groups were compared using the independent t-test or Mann-Whitney U test for continuous data and the chi-square test for categorical data. For comparisons among the three groups, a one-way analysis of variance, Wilcoxon test, or chi-square test was employed, as appropriate. Logistic regression analysis was used to analyze the factors influencing pregnancy outcomes and assess the association between IH and pregnancy outcomes. The Cochran-Armitage trend test was performed to analyze the effects of different degrees of IH on adverse pregnancy outcomes. Statistical significance was set at *P* < 0.05.

## Results

### Comparison of maternal characteristics in the first trimester

A total of 1236 pregnant women were included in this study. Among them, 109 (8.8%) were diagnosed with IH, while 1127 (91.2%) had normal thyroid function. Compared to women with euthyroidism, women with IH were younger, multiparous, had a lower rate of abnormal pregnancy history, and exhibited higher TSH and lower FT4 levels (*P* < 0.05). No significant differences were observed between the two groups in terms of BMI, FPG, TC, TG, HDL-C, LDL-C, Fe, and ferritin levels (*P* > 0.05) (**Table 1**).

**Table 1:** Comparison of maternal characteristics in the first trimester between the euthyroid group and the IH group.

	Euthyroid group (n =1127)	IH group (n =109)	*P*
**Age (years)**	30 (6)	31 (5)	0.827
**≥ 35**	216 (19.2%)	12 (11.0%)	0.036
**BMI (kg/m^2^)**	21.36 (3.50)	21.77 (3.64)	0.114
**≥ 24**	219 (19.4%)	27 (24.8%)	0.183
Parity			
**≥ 1**	394 (35.0%)	91 (83.5%)	**0.000**
**Abnormal pregnancy history**	169 (15.0%)	4 (3.7%)	**0.001**
**FPG (mmol/L)**	4.88 (0.50)	4.92 (0.34)	0.560
**TC (mmol/L)**	3.88 (0.80)	4.02 (0.97)	0.170
**TG (mmol/L)**	0.84 (0.51)	0.86 (0.46)	0.794
**HDL-C (mmol/L)**	1.40 (0.35)	1.42 (0.35)	0.083
**LDL-C (mmol/L)**	1.99 (0.70)	2.10 (0.62)	0.061
**Fe (mmol/L)**	7.82 (0.88)	7.62 (0.52)	0.281
**Ferritin (ng/ml)**	50.60 (45.00)	51.90 (43.35)	0.957
**FT4 (pmol/L)**	16.90 (2.60)	13.20 (1.15)	**0.000**
**TSH (uIU/ml)**	1.44 (1.16)	1.76 (1.18)	**0.001**

The bold values indicate P<0.05. IH Hypothyroxinemia, BMI Body mass index, FPG Fasting plasma glucose, TC Total cholesterol, TG Triglycerides, HDL-C High-density lipoprotein cholesterol, LDL-C Low-density lipoprotein cholesterol, Fe Ferrum, FT4 Free thyroxine, TSH Thyroid stimulating hormone.

Based on FT4 levels, 40 cases were categorized into the mild IH group, and 69 into the severe IH group. In both the mild and severe IH groups, the proportions of multiparous women and TSH levels were higher than in the euthyroid group (*P* < 0.05). Additionally, compared to the euthyroid group, the mild and severe IH groups had lower rates of abnormal pregnancy history and FT4 levels (*P* < 0.05). There were no significant differences in the mean age, BMI, FPG, TC, TG, HDL-C, LDL-C, Fe, or ferritin levels among the three groups (*P* > 0.05) (**Table 2**).

**Table 2:** Comparison of maternal characteristics in the first trimester among the euthyroid group, the mild IH group and the severe IH group.

	Euthyroid group (n (n=1127)	IH group		*P*
		Mild IH group (n =40)	Severe IH group (n =69)	
**Age (years)**	30 (6)	31 (4)	30 (5)	0.976
**≥ 35**	216 (19.2%)	5 (12.5%)	7 (10.1)	0.106
**BMI (kg/m^2^)**	21.36 (3.50)	21.56 (3.69)	22.00 (3.45)	0.285
**≥ 24**	219 (19.4%)	11 (27.5%)	16 (23.2)	0.355
Parity				
**≥ 1**	394 (35.0%)	34 (85.0%) *	57 (82.6) *	**0.000**
**Abnormal pregnancy history**	169 (15.0%)	2 (5.0%) *	2 (2.9) *	**0.005**
**FPG (mmol/L)**	4.88 (0.50)	4.91 (0.30)	4.93 (0.45)	0.843
**TC (mmol/L)**	3.88 (0.80)	4.14 (1.01)	4.00 (1.00)	0.367
**TG (mmol/L)**	0.84 (0.51)	0.84 (0.46)	0.88 (0.46)	0.927
**HDL-C (mmol/L)**	1.40 (0.35)	1.43 (0.40)	1.42 (0.32)	0.219
**LDL-C (mmol/L)**	1.99 (0.70)	2.05 (0.64)	2.12 (0.61)	0.164
**Fe (mmol/L)**	7.82 (0.88)	8.10 (0.04)	7.59 (0.55)	0.358
**Ferritin (ng/ml)**	50.60 (45.00)	57.30 (47.30)	51.60 (44.10)	0.947
**FT4 (pmol/L)**	16.90 (2.60)	13.60 (0.30) *	12.80 (1.05) *	**0.000**
**TSH (uIU/ml)**	1.44 (1.16)	1.74 (1.11) *	1.77 (1.23) *	**0.003**

Compared with the euthyroid group, *P < 0.05. The bold values indicate P<0.05.

IH Hypothyroxinemia, BMI Body mass index, FPG Fasting plasma glucose, TC Total cholesterol, TG Triglycerides, HDL-C High-density lipoprotein cholesterol, LDL-C Low-density lipoprotein cholesterol, Fe Ferrum, FT4 Free thyroxine, TSH Thyroid stimulating hormone.

### Comparison of pregnancy outcomes

**Figure 2:**
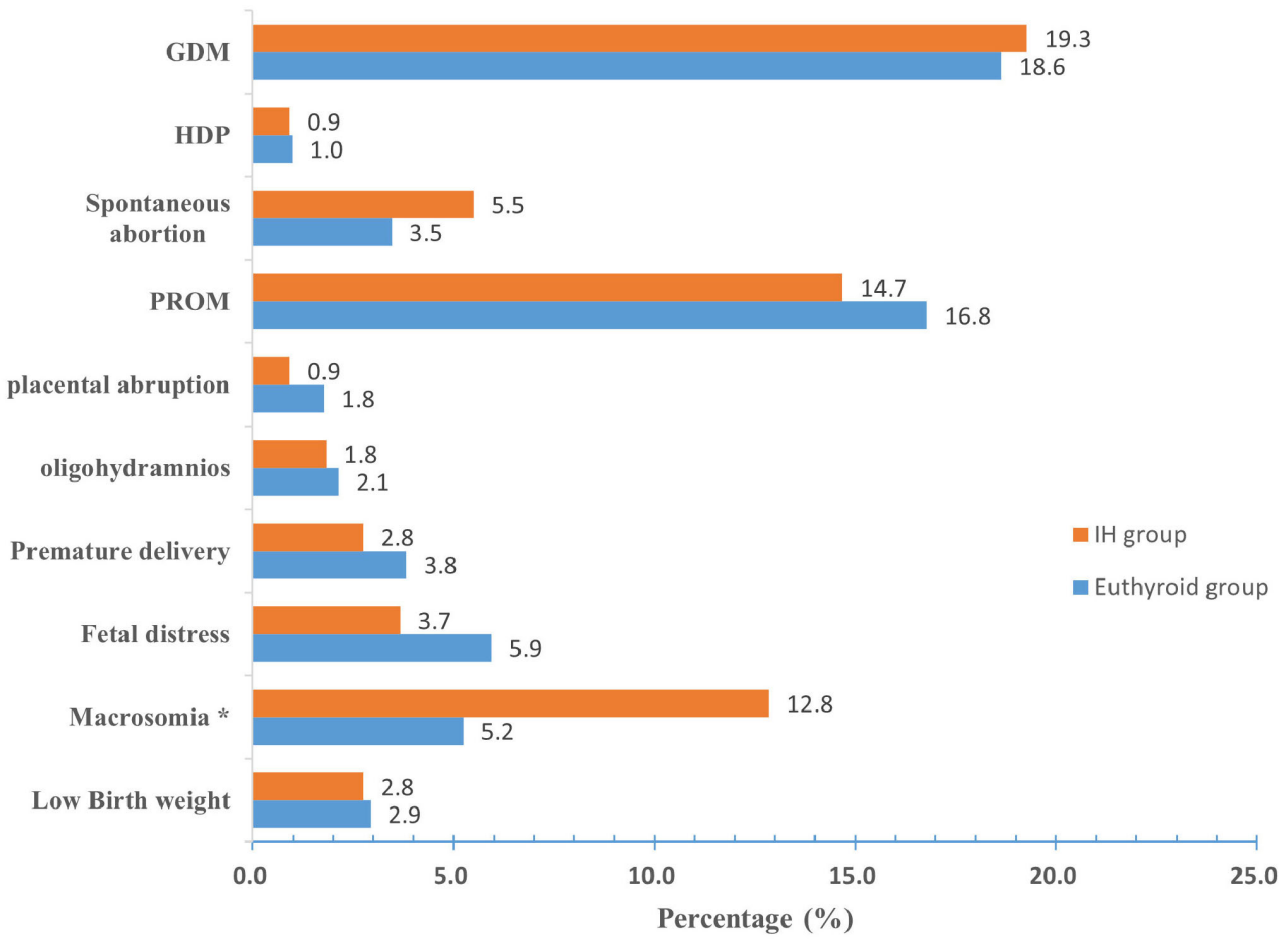
Prevalence of pregnancy outcomes in the euthyroid and IH groups. Compared with the euthyroid group, *P < 0.05. IH Hypothyroxinemia, GDM gestational diabetes mellitus, HDP hypertensive disorders of pregnancy, PROM premature rupture of membranes.

The prevalence of macrosomia was higher in the IH group than in the euthyroid group (12.8% vs. 5.2%, *P* < 0.05). Although the incidences of GDM and spontaneous abortion were higher in the IH group, the differences were not significant (*P* > 0.05). Furthermore, the incidence of HBP, PROM, placental abruption, oligohydramnios, premature delivery, fetal distress, or low birth weight was lower in the IH group than in the euthyroid group; however, these differences were not significant (*P* > 0.05) (**Figure 2**).

**Figure 3:**
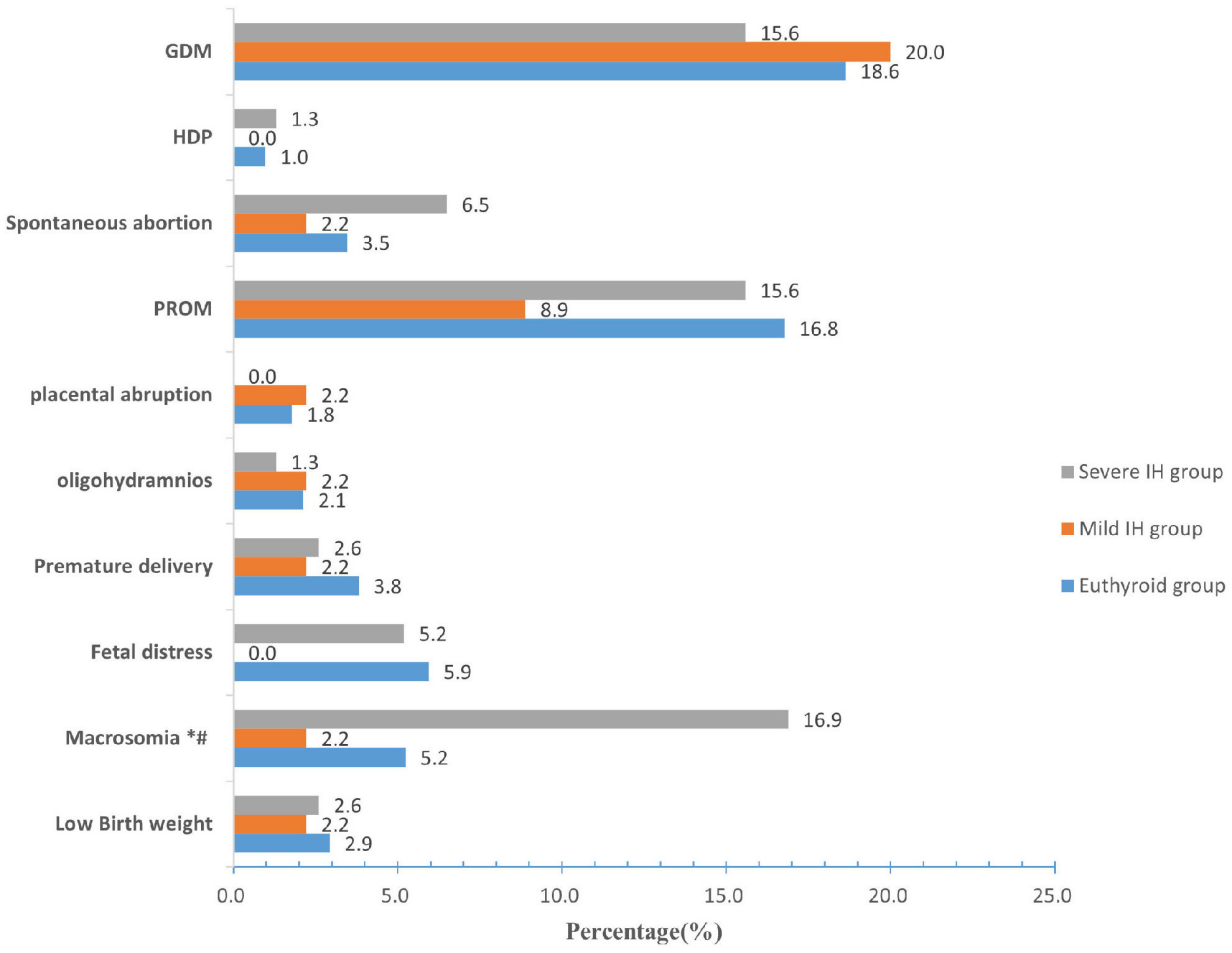
Prevalence of pregnancy outcomes among the euthyroid, mild IH, and severe IH groups. Compared with the euthyroid group, *P < 0.05; Compared with the mild IH group, ^#^ *P*< 0.05. IH Hypothyroxinemia, GDM gestational diabetes mellitus, HDP hypertensive disorders of pregnancy, PROM premature rupture of membranes.

The incidence of macrosomia in the euthyroid, mild IH, and severe IH groups was 5.2%, 2.2%, and 16.9%, respectively. Compared to women with euthyroid or mild IH, those with severe IH were more likely to give birth to macrosomia (*P* < 0.05). However, no significant differences were found in the incidences of GDM, HDP, spontaneous abortion, PROM, placental abruption, oligohydramnios, premature delivery, fetal distress, or low birth weight among the three groups (all *P* > 0.05) (**Figure 3**).

### Influencing factors of pregnancy outcomes

Univariate logistic regression analysis was employed to initially screen factors influencing adverse pregnancy outcomes during early pregnancy. The results revealed that GDM was associated with age ≥ 35 years, BMI ≥ 24 kg/m^2^, multiparity, FPG, and TC (all *P* < 0.05). HDP was associated with BMI ≥ 24 kg/m^2^ (*P* < 0.05). Miscarriage was associated with an abnormal pregnancy history (*P* < 0.05). PROM was associated with age ≥ 35 years and multiparity (*P* < 0.05). Macrosomia was associated with BMI ≥ 24 kg/m^2^, multiparity status, and TC levels (all *P* < 0.05). Low birth weight was significantly associated with FPG (*P* < 0.05) (**Table 3**).

**Table 3:** The influencing factors of pregnancy outcomes.

	age ≥ 35 years		BMI ≥ 24 kg/m^2^		multiparity		Abnormal-pregnancy history		FPG		TC	
	OR (95% CI)	*P*	OR (95% CI)	*P*	OR (95%CI)	*P*	OR (95% CI)	*P*	OR (95% CI)	*P*	OR (95% CI)	*P*
**GDM**	1.97 (1.41, 2.74)	**0.000**	2.28 (1.66, 3.15)	**0.000**	1.53 (1.15, 2.04)	**0.004**	1.22 (0.82, 1.81)	0.327	2.87 (2.00, 4.11)	0.000	1.37 (1.13, 1.66)	**0.001**
**HDP**	1.48 (0.40, 5.51)	0.559	4.10 (1.31, 12.82)	**0.015**	1.56 (0.50, 4.85)	0.447	2.07 (0.55, 7.71)	0.280	1.36 (0.81, 2.29)	0.242	0.92 (0.41, 2.07)	0.839
**Spontaneous abortions**	1.84 (0.95, 3.57)	0.070	1.01 (0.48, 2.12)	0.987	0.94 (0.51, 1.73)	0.838	2.06 (1.02, 4.14)	**0.044**	1.26 (0.85, 1.87)	0.243	1.16 (0.78, 1.73)	0.459
**PROM**	0.54 (0.34, 0.85)	**0.007**	0.77 (0.52, 1.14)	0.194	0.69 (0.50, 0.95)	**0.024**	0.66 (0.41, 1.07)	0.092	0.95 (0.68, 1.31)	0.743	0.88 (0.71, 1.09)	0.246
**Placental abruption**	1.79 (0.69, 4.66)	0.234	1.26 (0.46, 3.48)	0.652	1.42 (0.60, 3.36)	0.430	0.64 (0.15, 2.78)	0.554	1.35 (0.87, 2.10)	0.177	1.22 (0.69, 2.14)	0.489
**Oligohydramnios**	0.57 (0.17, 1.92)	0.365	0.73 (0.25,2.13)	0.561	0.97 (0.44, 2.15)	0.935	1.12 (0.38, 3.29)	0.837	0.64 (0.24, 1.71)	0.371	1.04 (0.61, 1.78)	0.879
**Premature delivery**	1.41 (0.71, 2.82)	0.332	1.62 (0.84, 3.12)	0.151	1.44 (0.80, 2.60)	0.226	0.92 (0.38, 2.20)	0.849	0.99 (0.54, 1.83)	0.975	1.35 (0.93, 1.94)	0.112
**Fetal distress**	0.80 (0.41, 1.55)	0.509	1.29 (0.73, 2.26):	0.381	0.59 (0.35, 1.00)	0.051	2.40 (1.38, 4.17)	**0.002**	1.00 (0.62, 1.63)	0.991	0.84 (0.59, 1.19)	0.323
**Macrosomia**	0.96 (0.52, 1.77)	0.885	2.70 (1.65, 4.42)	**0.000**	1.38 (1.01, 2.21)	**0.048**	0.97 (0.49, 1.94)	0.940	1.21 (0.85, 1.73)	0.287	1.35 (1.01, 1.81)	**0.048**
**Low birth weight**	1.07 (0.46, 2.47)	0.876	1.81 (0.88, 3.72)	0.109	0.87 (0.44, 1.74)	0.697	0.99 (0.38, 2.58)	0.985	1.54 (1.06, 2.25)	0.024	1.24 (0.81, 1.89)	0.324

The bold values indicate P<0.05. GDM gestational diabetes mellitus, HDP hypertensive disorders of pregnancy, PROM premature rupture of membranes, BMI Body mass index, FPG Fasting plasma glucose, TC Total cholesterol.

### Association of IH in the first trimester with adverse pregnancy outcomes

Results from logistic regression analysis, with adverse pregnancy outcomes as the dependent variable and IH as an independent variable, showed that IH in the first trimester increased the risk of macrosomia in all models (crude OR: 2.67, 95% CI: 1.44-4.96, *P* = 0.002; adjusted OR: 2.32, 95% CI: 1.13-4.75, *P* = 0.022). However, no significant relationship was found between IH in the first trimester and GDM, HDP, spontaneous abortion, PROM, placental abruption, oligohydramnios, premature delivery, fetal distress, or low birth weight (all *P* > 0.05).

Furthermore, we observed a striking association between a higher risk of macrosomia and more severe IH using the Cochran-Armitage trend test (*P*_trend_ < 0.05) (**Table 4**).

**Table 4:** Association of IH in the first trimester with adverse pregnancy outcomes.

		Euthyroid group		Mild IH group		Severe IH group		*P*_trend_
		OR (95% CI)	*P*	OR (95% CI)	*P*	OR (95% CI)	*P*	
**GDM**	Model 1	1.04 (0.63, 1.72)	0.872	1.27 (0.60, 2.70)	0.539	0.92 (0.49, 1.74)	0.797	0.968
	Model 2	1.06 (0.61, 1.84)	0.829	1.19 (0.53, 2.65)	0.673	0.99 (0.49, 1.96)	0.965	0.933
**HBP**	Model 1	0.94 (0.12, 7.35)	0.952					
	Model 2	0.87 (0.10, 7.74)	0.900					
**Spontaneous abortion**	Model 1	1.63 (0.67, 3.93)	0.281	0.72 (0.10, 5.34)	0.744	2.18 (0.83, 5.72)	0.113	0.163
	Model 2	2.15 (0.76, 6.09)	0.148	1.07 (0.14, 8.33)	0.951	2.90 (0.92, 9.10)	0.069	0.085
**PROM**	Model 1	0.85 (0.49, 1.48)	0.575	0.55 (0.19, 1.57)	0.264	1.05 (0.55, 1.99)	0.893	0.810
	Model 2	1.03 (0.57, 1.86)	0.931	0.66 (0.23, 1.91)	0.439	1.27 (0.64, 2.52)	0.496	0.675
**Placental abruption**	Model 1	0.51 (0.07, 3.86)	0.516					
	Model 2	0.49 (0.06, 3.95)	0.502					
**Oligohydramnios**	Model 1	0.86 (0.20, 3.69)	0.838	1.18 (0.16, 8.93)	0.874	0.68 (0.09, 5.07)	0.703	0.761
	Model 2	0.86 (0.19, 4.02)	0.852	1.16 (0.14, 9.39)	0.889	0.69 (0.09, 5.49)	0.724	0.775
**Premature delivery**	Model 1	0.71 (0.22, 2.34)	0.577	0.65 (0.09, 4.82)	0.670	0.75 (0.18, 3.17)	0.699	0.616
	Model 2	0.60 (0.17, 2.08)	0.422	0.50 (0.07, 3.88)	0.508	0.67 (0.15, 2.94)	0.593	0.483
**Fetal distress**	Model 1	0.60 (0.22, 1.69)	0.335					
	Model 2	0.96 (0.32, 2.87)	0.948					
**Macrosomia**	Model 1	2.67 (1.44, 4.96)	**0.002**	0.46 (0.06, 3.44)	0.452	4.20 (2.18, 8.11)	**0.000**	**0.000**
	Model 2	2.32 (1.13, 4.75)	**0.022**	0.38 (0.05, 2.91)	0.350	3.89 (1.82, 8.29)	**0.000**	**0.002**
**Low birth weight**	Model 1	0.94 (0.28, 3.11)	0.917	0.85 (0.11, 6.38)	0.874	0.99 (0.23, 4.21)	0.989	0.949
	Model 2	1.14 (0.31, 4.14)	0.842	0.98 (0.12, 7.78)	0.982	1.24 (0.27, 5.71)	0.778	0.799

Model 1: none (univariable); Model 2: adjusted for age, BMI, parity, abnormal pregnancy history, FPG and TC. The bold values indicate P<0.05.

IH Hypothyroxinemia, GDM gestational diabetes mellitus, HDP hypertensive disorders of pregnancy, PROM premature rupture of membranes, BMI Body mass index, FPG Fasting plasma glucose, TC Total cholesterol.

## Discussion

IH is a common form of thyroid dysfunction observed in the pregnant population (16). Previous studies have reported a wide range in the incidence rate of IH in pregnant women, ranging from 1.3% to 23.9% (17), with the most commonly reported rates being 8-10% (18). In our study, IH was found in 8.8% of the pregnant women in their first trimester. The differences in the prevalence of IH across studies may be attributed to variations in diagnostic criteria, gestational age of the participants, iodine nutritional status of the study population, and other factors.

Studies examining the relationship between IH and adverse pregnancy outcomes remain limited and controversial (10). Casey et al. (19) analyzed a large cohort of 17,298 pregnant women during the first half of pregnancy and reported no significant differences in the prevalence of placental abruption, preterm birth, neonatal asphyxia, or pregnancy-induced hypertension between the IH and euthyroid groups. Similarly, a retrospective study involving 7051 pregnant women showed that IH in the first trimester did not increase adverse outcomes, including preterm birth, gestational hypertension, preeclampsia, GDM, placental abruption, PROM, intrauterine growth restriction (IUGR), polyhydramnios, stillbirth, or small for gestational age (15). Chen et al. (20) also demonstrated that IH during early pregnancy was not associated with placenta previa, placental abruption, preterm birth, fetal distress, intrauterine fetal death, low birth weight, GDM, HDP, IUGR, or PROM. These findings are consistent with our study, where we found no significant association between IH in the first trimester and GDM, HDP, spontaneous abortion, PROM, placental abruption, oligohydramnios, premature delivery, fetal distress, or low birth weight. However, some meta-analyses (10, 16) have reported a significantly increased risk of GDM, PROM, preterm birth, and fetal distress in pregnant women with IH compared to that in women with euthyroidism. These discrepancies could be attributed to differences in the study population, including ethnicity and gestational age, and variations in thyroid function testing methods and diagnostic criteria.

Thyroid hormones are crucial in promoting and maintaining fetal growth and development by regulating metabolism (21), including fetal glucose, protein, and fat, and oxygen consumption and other cofactors that directly affect bone growth, tissue differentiation, and proliferation (22). Although the thyroid gland is the first endocrine organ involved in fetal development, it does not begin to secrete thyroid hormones until approximately 18-20 weeks of gestation (23). Therefore, nearly all thyroid hormones required by the fetus during the early stages of pregnancy depend on the maternal supply (24). Maternal thyroid hormone deficiency can significantly affect fetal growth and development, especially during early pregnancy. Previous studies have shown that maternal FT4 levels in the first trimester are negatively associated with neonatal birth weight (25, 26). Thus, IH during early pregnancy is an independent factor for macrosomia (27, 28). Consistently, in the present study, the prevalence of macrosomia was higher in the IH group than in the euthyroid group (12.8% vs. 5.2%).

The results of the univariate logistic regression analysis revealed a correlation between IH during early pregnancy and the occurrence of macrosomia (crude OR: 2.67, 95% CI: 1.44-4.96, *P* = 0.002). After adjusting for confounding factors such as age, BMI, parity, abnormal pregnancy history, FPG, and TC, the relationship remained statistically significant (adjusted OR: 2.32, 95% CI: 1.13-4.75, *P* = 0.022). These results were consistent with those of previous studies (10, 16). Furthermore, our results indicated an increasing trend in the risk of macrosomia with IH severity (crude *P*_trend_ = 0.000; adjusted *P*_trend_ = 0.002).

The mechanism underlying maternal IH-mediated effects on macrosomia may involve the reduction of FT4 transfer from the placenta to the fetus, leading to slower fetal metabolism and subsequent weight gain (29, 30). Another possible reason is that lower FT4 levels can cause elevated maternal circulating glucose, resulting in increased placental transfer of glucose to the fetus and promoting higher birth weight (26, 28). In our study, the mean FPG level of pregnant women with IH was higher than that of those with euthyroidism (4.92 mmol/L vs. 4.88 mmol/L), although the difference was not statistically significant. Whether LT4 intervention reduces the occurrence of macrosomia in pregnant women with IH remains unclear. Gong et al. (31) reported a lower incidence of macrosomia in women with IH who received LT4 treatment at an average gestational age of 6.27 weeks than in those who did not receive LT4 treatment (8.42% vs. 14.15%). While these differences did not achieve statistical significance, they may still hold clinical relevance that warrants further investigation. In addition, the initial timing of LT4 therapy is crucial for improving pregnancy outcomes (17). Therefore, more randomized controlled trials are required to investigate the impact of LT4 intervention on the risk of macrosomia in women with IH during the first trimester.

This study has several strengths. First, this was a prospective cohort study that investigated the relationship between IH during early pregnancy and adverse pregnancy outcomes, which enhanced the reliability of the findings. Second, when analyzing the relationship, we adjusted for potential confounding factors to improve the accuracy of the results. Third, we analyzed the effect of different degrees of IH severity on adverse pregnancy outcomes, providing evidence for the stratified management of IH in women in the first trimester. However, this study has some limitations. First, owing to the single-center design, the generalizability of our findings may be limited. Second, we did not measure the iodine intake in pregnant women. Nevertheless, all participants in our study had resided for > 5 years in Beijing, which is considered an iodine-sufficient region.

## Conclusions

Our study found that IH during the first trimester was associated with an increased risk of macrosomia, particularly in patients with more severe IH. However, we did not observe an association between IH in the first trimester and any other studied outcomes, including GDM, HDP, spontaneous abortion, PROM, placental abruption, oligohydramnios, premature delivery, fetal distress, or low birth weight. Our findings contribute to a better understanding of the potential risks associated with IH, particularly regarding macrosomia. Furthermore, our research delves into the degree of IH severity and its effect on pregnancy outcomes, providing valuable evidence for stratified IH management in early pregnancy. The potential role of LT4 intervention in reducing the risk of macrosomia requires further investigations across multiple centers, and will be the focus of our future research.

## Data availability statement

The raw data supporting the conclusions of this article will be made available by the authors, without undue reservation.

## Ethics statement

The studies involving humans were approved by the Ethics Committee of Peking University International Hospital. The studies were conducted in accordance with the local legislation and institutional requirements. The participants provided their written informed consent to participate in this study.

## Author contributions

JD: Conceptualization, Investigation, Methodology, Software, Visualization, Writing – original draft, Writing – review & editing. LJ: Conceptualization, Methodology, Project administration, Resources, Supervision, Visualization, Writing – review & editing. XZ: Conceptualization, Funding acquisition, Methodology, Project administration, Resources, Supervision, Writing – review & editing. NY: Data curation, Investigation, Writing – review & editing. JS: Data curation, Investigation, Writing – review & editing. DZ: Data curation, Investigation, Writing – review & editing.
